# Effect of Parity on Reproductive Performance and Composition of Sow Colostrum during First 24 h Postpartum

**DOI:** 10.3390/ani10101853

**Published:** 2020-10-12

**Authors:** Mónica Segura, Silvia Martínez-Miró, Miguel José López, Josefa Madrid, Fuensanta Hernández

**Affiliations:** Department of Animal Production, Faculty of Veterinary, Regional Campus of International Excellence “Campus Mare Nostrum”, University of Murcia, 30100 Murcia, Spain; monicamarcela.segurar@um.es (M.S.); mjlopeza@um.es (M.J.L.); alimen@um.es (J.M.); nutri@um.es (F.H.)

**Keywords:** sows, colostrum, immunoglobulin, piglets, birth weight

## Abstract

**Simple Summary:**

This work looks at the effects of the number of parturitions on the reproductive performances of sows and on the composition of the colostrum. We also studied the effect that sample collection time (0–24 h postpartum) has on colostrum composition. The reproductive parameters of the sows were hardly affected by the number of parturitions, and only the birth weights of piglets born alive were lower in sows first, third and fifth farrowing. The colostrum of primiparous sows had higher concentrations of dry matter, fat, lactose and non-fat-solids than those of the rest of the sows. The postpartum sampling time had a strong effect on colostrum composition, the first sample being higher in protein and lower in lactose than those obtained later. From an immunological point of view, the concentration of immunoglobulins in the colostrum was not influenced by the sow’s parity. Serum IgG showed a strong correlation with colostrum IgG and IgM, and colostrum IgG with colostrum IgM, but not with IgA. In turn, IgA did not correlate with any other immunoglobulin, which suggests that the immunoglobins in colostrum behave differently.

**Abstract:**

The aim of this study is to assess the effects of parity number on sow reproductive performance and the chemical and immunological composition of colostrum and immunoglobin concentrations in the sera of the sows. Colostrum samples were collected at 0, 6 and 24 h after the births of the first piglets from 56 sows with different numbers of parturitions (ranging 1–6). The piglets born alive to primiparous sows had lower birth weights (*p* < 0.05) than piglets from second and fourth parturition sows. The colostrum composition was influenced (*p* < 0.05) by parity number: primiparous sows had higher concentrations of dry matter, fat, lactose and non-fat-solids. No parity-dependent differences were found concerning total protein amount. Colostrum composition was drastically affected (*p* < 0.001) by sampling time—the highest concentrations of dry matter and protein and lowest concentrations of fat and lactose were found immediately after parturition (0 h). The study revealed no effect of parity (*p* ≥ 0.05) on the concentrations of immunoglobulins in colostrum. The immunoglobulin with the highest level in sow serum at day 110 of gestation was IgG, while IgA showed the lowest values and greater variability with respect to parity from an immunological point of view. Regarding the relationship between serum Ig levels at the end of gestation and colostrum Ig, serum IgG showed a strong correlation with colostrum IgG and IgM, while colostrum IgG was strongly related with colostrum IgM, but not with IgA. IgA did not correlate with any other immunoglobulin. The different behaviors of the immunoglobins in colostrum were probably due to IgG coming almost exclusively from the sows’ sera, whereas IgA is mainly synthetized by the mammary gland.

## 1. Introduction

Colostrum is a nutrient-rich liquid secreted by the mammary gland of mammals after giving birth and during the first 24–48 h postpartum, before changing to mature milk [1]. Colostrum and milk production by the sow are primary limiting factors affecting the survival, growth and development of newborns. Both secretions are complex biological fluids that contain a number of nutrients as well as protective factors, such as macro- and micronutrients, vitamins and bioactive substances (immunoglobulins, enzymes and growth factors), which play an important role in early gastrointestinal development [2].

Many components of colostrum change with time and, in terms of composition, can be divided into three phases: early (0 h), mean (12 h) and late (24 h) colostrum [3]. The transition from colostrum to milk occurs rapidly between 24 and 36 h after the onset of farrowing. Compared to mature milk, sow colostrum is characterized by a lower concentration of lactose (3% to 5%) and fat (5% to 8%), but a higher percentage of dry matter (18% to 28%) and protein (5% to 17%) [1,3]. In addition, the concentrations of immunoglobulins (IgG, IgA and IgM) are higher in colostrum than in milk, and these isotypes in porcine milk are identical to those in blood serum [1]. The main protein component of pig colostrum is IgG, decreasing from more than 80% in early colostrum to much lower values in mature milk. Hurley [1] showed that the IgG content of the colostrum decreased to 4.8% of its original concentration at 72 h post farrowing and Frenyo et al. [4] found that it decreased to 3.2% of its original concentration at the fifth day of lactation. Colostrum also has more bioactive components [5], while milk contains more casein and serum proteins [2,5]. The first secretions from the mammary gland after farrowing are largely absorbed by piglets and appear in their blood within a few hours of secretion from the udder [1].

Previous studies have reported that macronutrient levels and total immunoglobulin concentrations in colostrum and sow milk vary among breeds and are influenced by many factors, such as nutrition, parity number, endocrine control, mammal health status and environmental conditions [6,7]. Differences have also been found according to the experimental design and management, including vaccination, the use of oxytocin, the udder section sampled and the time after farrowing that the colostrum sample is collected, which is an important limiting factor in order to understand the colostrum composition. The content of some colostrum and milk components, including fat, fat-soluble vitamins, some minerals, fatty acids and immunoglobins, can be affected by these factors [1,6,8]. From a practical point of view, it would be very interesting to know if sows with different parity numbers show differences in colostrum composition, since the adoption of piglets during the first hours of life is a common practice in pig farms. Thus, the weakest piglets could be adopted by sows with colostrum of higher nutritional or immunological qualities. However, little information is available about the effect of sow parity number on colostrum composition. In addition, although IgG is the major Ig in colostrum, IgA and IgM are also important for the complete immunological protection of the piglet and few studies look at the factors that can affect their concentrations in colostrum. Among the factors that can affect the concentration of immunoglobulins of colostrum is the immunological status of the sow at the end of pregnancy. Curtis and Bourne [9] showed that all colostral IgG and a high proportion of colostral IgM appear to be derived from serum, and Quesnel et al. [10] concluded that IgG concentrations in maternal blood in late pregnancy explain 36% of the variability observed in IgG concentrations in colostrum at the onset of farrowing. In light of this, in this work, we wanted to study the relationship between the sow serum immune status and the colostrum immune concentration. Therefore, the objective of the present experiment was to evaluate the effect of parity and time of collection on sow reproductive performance and colostrum composition during the first 24 h postpartum, with special emphasis on the immune profile. Additionally, the effect of sow parity number on serum immunoglobin concentration at the end of gestation was studied.

## 2. Materials and Methods

### 2.1. Animals, Measurements and Experimental Design

Experimental procedures and animal handling practices were in accordance with the European Union guidelines for the care and use of research animals (2010/63/EU Directive) and were approved by the Ethics Committee of Animal Experimentation of the University of Murcia and the Autonomous Community of the Region of Murcia (A-13170805).

The experiment was performed at a commercial farm in Spain in a one-week batch management system (mean parity: 2.9, range: 1–6). A total of 56 crossbred sows (Large-White × Landrace), 20 primiparous and 36 multiparous, distributed in groups of nine according to the number of parities (2nd, 3rd, 4th and ≥ 5th) were included in this study. The sows were moved to the farrowing rooms at 110 d of gestation and were housed in individual farrowing crates (2.0 × 2.5 m) on a slatted plastic floor. Sows had free access to water and were fed twice daily on a standard gestation diet based on barley and soy-bean meal. The diets were formulated following the recommendations of the Spanish Foundation for the Development of Animal Nutrition (FEDNA) [11]. The body conditions of the sows were assessed both by visual appraisal (score 1 to 5; 1 = extremely thin, 5 = extremely fat) and by means of back-fat and loin depth measurements using the P2 method at day 110 of gestation [12]. Ultrasound measurements were performed using ultrasound scan equipment with a linear probe (SF-1 Wireless Backfat and Loin Depth Scanner, (Sonivet, Beijing, China), which is a B-mode ultrasound device equipped with a 45 mm, 5.0-MHz linear array transducer. The images were obtained taking two measurements 6.5 cm from the dorsal midline at the level of the last rib, using the average of the measurements for further calculations. On the day of farrowing, the parturition process was supervised, but any interference with the process was minimized. Immediately after birth, the time of birth of the first piglet was recorded, and each piglet was dried, their umbilical cords were clamped, and they were weighed using electronic scales. The following reproductive variables of sows and their litters were recorded: parity number, gestation length, body condition score, back-fat thickness, loin depth, number of piglets born alive per litter (NBA), number of piglets born dead per litter (NBD), live piglet birth weight (PBW) and total litter birth weight (LBW).

### 2.2. Samples Collection

A blood sample (about 5 mL) was collected at day 110 of gestation from the jugular vein of each sow, and placed in vacuum tubes without additives (Vacuette^®^, Greiner Bio-One, Kremsmünster, Austria) and centrifuged at 2000× *g* for 10 min. The serum samples were stored at −80 °C until analysis.

**Figure 1:**
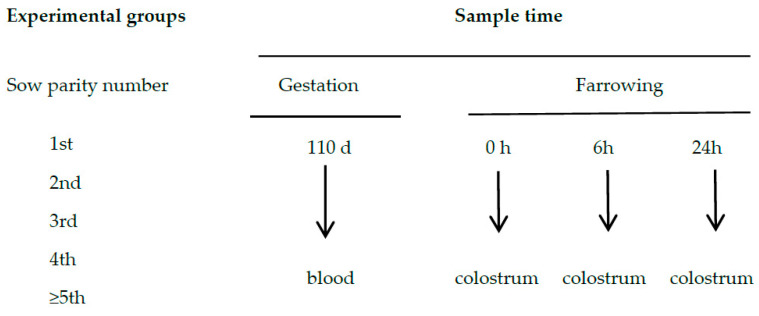
Experimental groups and sampling times for the collection of blood and colostrum.

During the first 24 h of lactation, counting from the first piglet’s birth, hand-milked colostrum (20 mL) was obtained from each sow at three times: immediately after the birth of the first piglet (T0), and six hours (T6) and twenty-four hours (T24) after the onset of parturition [13] (Figure 1). These samples were taken from the anterior, middle and posterior teats of the udder and then pooled. Oxytocin (10 IU/animal, Facilpart, SYVA Laboratories, León, Spain) was used at T6 and T24 to induce colostrum ejection and facilitate its collection. For proper hygiene, the udder of each animal was washed with warm water and dried before collecting samples. Individual colostrum samples (2 mL, twice) were aliquoted to determine immunoglobulin concentration and stored at −80 °C until analysis. The remaining colostrum samples were preserved immediately with azidiol (4 μL/mL) to inhibit bacterial growth, according to [14], and stored at 4 °C until the chemical composition was assessed (up to 48 h later).

### 2.3. Chemical Composition

The fat, total protein, lactose and total solids contents and the somatic cell counts of colostrum samples were determined using a MilkoScan FT6000 Analyzer (Foss Electric, Hillerød, Denmark), comprising a Fossomatic6000 somatic cell counter. Prior to analysis, samples were diluted with distilled water to ensure that the sample composition fell within the validated calibration range for the infrared analyzer. The regression equations were validated for sow milk: using the Kjeldahl method for the protein content [15], the international gravimetric basic Rose Gottlieb method for total fat [15], and the drying method for the dry matter content while the lactose content was calculated by difference using the following formula: %lactose = %total solids − (%protein + %fat + %ash) [16].

### 2.4. Immunological Parameters

The total concentrations of immunoglobulins in colostrum and serum samples were measured by indirect enzyme linked immunosorbent assay (ELISA) kits for specific commercial pig IgG, IgA and IgM (Bethyl Laboratories, Inc., Montgomery, TX, USA; references E101-104, E101-117 and E101-102) by following the manufacturer’s instructions. The absorbance at 450 nm was measured using a microplate reader (Infinite M200PRO, Tecan Trading AG, Männedorf, Switzerland).

### 2.5. Data Analysis

Statistical analyses were carried out using SPSS Statistics 15.0 software (IBM SPSS, Chicago, IL, USA). Data from all sows were used—20 primiparous and 36 multiparous distributed in groups of 9 according to the parity numbers (2nd, 3rd, 4th and ≥ 5th). All numerical data are presented as LS means ± SEM, and differences are considered as statistically significant at the 95% confidence level (*p* < 0.05). The differences between means were determined by Fisher’s LSD test. An analysis of variance was performed to study the effect of the sow’s parity number on the reproductive performance variables analyzed (gestation length, sow body condition, back-fat thickness, loin depth, NBA, PBW and LBW), and serum IgG, IgM and IgA concentration in the sows, using a General Linear Model procedure with treatment as the fixed factor. The macronutrient components and IgG, IgA and IgM concentration in colostrum at T0, T6 and T24 were analyzed by a multivariate analysis (General Linear Model) with sow parity, time and their interaction as fixed factors. Pearson’s correlation coefficient (r) was used to determine linear relationships between mean colostrum and serum Ig concentrations, considering a significance level of *p* < 0.05.

## 3. Results

### 3.1. Reproductive Performance of Sow

The duration of gestation ranged from 114.4 to 115.0 d and averaged 114.7 ± 0.12 d. At day 110 of pregnancy, sow back-fat and loin depth averaged 18.1 ± 0.81 mm and 43.2 ± 1.12 mm, respectively (Table 1). No significant effect (*p* ≥ 0.05) of parity was observed on the case of back-fat thickness, loin depth, gestation length or body condition score in sows at day 110 of gestation. Similarly, there were no effects (*p* ≥ 0.05) of parity on NBA, NBD and LBW. Parity had an effect (*p* < 0.05) on individual PBW. While the mean PBW was 1.5 ± 0.03 kg, it was lower in primiparous sows (1.3 ± 0.05 kg) than in the progenies of the second or fourth parities (1.6 ± 0.06 kg and 1.5 ± 0.07 kg), but similar to that of the third or fifth parities (1.4 ± 0.07 kg and 1.4 ± 0.07 kg).

### 3.2. Colostrum Chemical Composition

The mean colostrum composition of sows with different parity number from the birth of the first piglet to 24 h after parturition is shown in Table 2. The DM content of colostrum secretions during the first 24 h was affected by parity and the time after parturition (*p* < 0.001). Colostrum from primiparous sows had the highest DM content, which slowly decreased from 0 to 24 h postpartum (as in all sows, irrespective of the number of parities). No parity effect (*p* ≥ 0.05) was detected on the protein content of sow colostrum, which also decreased rapidly from birth to 24 h postpartum (*p* < 0.001). However, the colostrum fat content differed with parity number (*p* < 0.05) and time (*p* < 0.001). The highest content of fat was found in the colostrum obtained from sows undergoing their first, second and fourth parities, and at 24 h. The values of lactose content were affected by parity (*p* < 0.05) and time (*p* < 0.001). The lactose content was highest in colostrum secretions from primiparous and fourth parity sows. Moreover, the lactose concentration increased with time since farrowing, the highest values occurring at 24 h postpartum. The total non-fat solids (NFS) of colostrum was affected by parity (*p* < 0.05) and sampling time (*p* < 0.001), the highest values being obtained in the colostrum from the first and fifth parity sows and at 0 h postpartum. There were no effects of parity and time postpartum on the colostrum somatic cell count (SCC) levels. No statistical interactions (*p* ≥ 0.05) were detected between parity number and sample collection time with regard to the chemical composition of colostrum from sows.

### 3.3. Immunological Parameters

The serum concentrations of immunoglobulin at 110 d gestation are shown in Table 3. The mean serum IgG and IgM concentrations in sows at this time were 21.9 ± 1.36 and 9.8 ± 0.42 mg/mL, respectively, and were not influenced by parity number (*p* ≥ 0.05). The mean value of serum IgA was 1.5 ± 0.12 mg/mL, the sows with ≥ 5 parities had the highest concentration (*p* < 0.05), but they only showed a statistical difference from the concentrations of fourth parity sows.

The concentrations of immunoglobulins in colostrum were not influenced (*p* ≥ 0.05) by the sows’ parities (Table 4). Colostrum IgG, IgM and IgA levels decreased (*p* < 0.01) from birth to 24 h after farrowing. In the case of IgG and IgA content, the decrease was noticeable at 6 h, while the decrease in colostrum IgM content was only observed at 24 h. No interactions (*p* ≥ 0.05) were detected between parity number and sample collection time with regard to colostrum immunoglobulin IgG, IgM and IgA composition.

Regression analysis revealed a positive association between sow serum IgG concentrations and the average colostrum IgG concentrations (*p* < 0.001), and also between the sow’s serum or colostrum IgG concentration and colostrum IgM (*p* < 0.01; *p* < 0.001, respectively) (Table 5).

## 4. Discussion

Colostrum is a source of important nutrients, such as protein, minerals and energy for growth, and aids the thermoregulation of suckling piglets, while providing them with passive immunity to protect them from pathogens until their own immune system develops [17]. The colostrum period is very short (0–24 h postpartum), and the content of many colostrum components changes substantially and rapidly, so that a high colostrum intake is a key factor for survival and growth during the early suckling period [18]. Some authors have proposed that sow parity, live-weight and back-fat depth can be used as indicators of reproductive performance and litter characteristics at birth and weaning [19,20].

In the present study, variables related to sow and litter characteristics were analyzed, in order to ascertain whether they are influenced by parity number. It has previously been shown that gilts produce litters with less variation in PBW [19] and lower growth rates [21] compared with middle-aged or older sows. Our results show that piglets born to primiparous sows had lower PBW, but did not differ in PBW compared to piglets of third or fifth parturition sows. There was a significant increase in PBW between the first and second parity, probably due to an increase in uterine capacity, as other studies have mentioned [22]. Nuntapaitoon et al. [7] found that primiparous sows had lower PBW than older sows, although they did not distinguish between second, third, fourth and fifth parity sows, as in our study. There were no parity differences for any of the other reproductive performance variables analyzed, which agrees with that reported by Carney-Hinkle et al. [15] who compared sows farrowing at the same time, as we did in our research. Lavery et al. [19] found that greater back-fat depth in late gestation was associated with a decrease in the number of piglets born alive but at a heavier body weight. However, in our study all sows, regardless of parity number, had similar back-fat thickness, loin depth and body condition score, which could explain our results concerning litter parameters.

This work also aimed to evaluate the effect of parity number on colostrum composition. The total solids content is significantly related to the fat and protein content of mammary secretions [23], and the differences found in DM content are probably due to diet, water intake or the individual sow’s health [24]. The concentration of DM in the colostrum samples, which was influenced by parity number, was higher in colostrum from primiparous sows compared with multiparous sows, in agreement with the preliminary studies [1,24].

At birth, piglets have limited body fat reserves and a low capacity to oxidize protein, colostrum fat and lactose, which are the main sources of energy for the newborn piglet [3]. It is well documented that fat is the most variable component in sow colostrum [17,18], which was confirmed in the present study. Several studies have also found that colostrum fat is highest in primiparous sows and declines as parity increases [18,25]. In the current study, although primiparous sows had the highest fat content (8%), we found no statistical difference in fat concentration between first, second and fourth parity sows. The third and fifth parity sows had a 16% lower colostrum fat content compared with the average of the other parities. Although other authors found no effect of parity on colostrum fat content [26,27], these discrepancies may be due to other factors. In fact, Declerck [28] showed that the relative contribution of parity on variations in fat levels was only 17%, with other factors, such as breed, having a greater influence. Interestingly, Luise et al. [27] found that the parity order did not affect the concentration of total lipid of colostrum, but it affected the different colostrum fatty acids. They also found a strong effect of the breed on the lipid composition of colostrum. The least variable component in colostrum is lactose, an important carbohydrate that is a rapidly assimilated energy compound for newborns [3]. Lactose concentration in sow colostrum was the highest (*p* < 0.05) in primiparous and fourth parity sows. Unlike in our results, other authors concluded that lactose variation in colostrum was due to dietary and endocrine factors rather than factors related to the sow, litter and parturition [28].

The current study found no parity differences in terms of total protein, which is largely consistent with the findings of other authors [28,29,30], who all found that protein levels were independent of sow parity. On the other hand, total NFS in colostrum was affected by parity as a result of the values obtained for lactose. A negative correlation has been described between parity and colostrum lactose content [28]. In contrast, a major colostrum lactose content in gilts at 48 h post farrowing has been noted [29]. Mammary gland physiology, hormones, vaccinations, management, nutritional status of sows and breed have all been reported as factors influencing lactose in colostrum [8,10,13,31]. In our study, in multiparous sows, no effect of parity on dry matter, protein and lactose colostrum content was found, in agreement with Nuntapaitoon et al. [7]. Therefore, the results suggest that multiparous sows produced a nutritionally similar colostrum, which is important in order to carry out piglet adoptions in the early postpartum hours.

Previous studies have described lower colostrum yield from gilts compared with older sows [7,11], so most colostrum components are expected to be more concentrated. In addition, due to the fact that some colostrum compounds are derived from the sow’s blood constituents, the use of body fat and protein reserves during late gestation is considered a possible factor that may partly explain the parity effect on variations in colostrum production [32,33]. This is probably due to primiparous sows prioritizing the destination of nutrients during lactation toward their maternal protein reserves, rather than colostrum yield [7,34].

The nutritional composition of colostrum was drastically affected by the stage of lactation. The main changes occurred during the first 6 h postpartum, as reported previously [17]. It is well known that all of these changes occur at the same time as the transition phase from colostrum to milk and the stabilization of mature milk [3].

The highest DM content was observed at T0 and then decreased by about 21% during the first 24 h after farrowing, in agreement with previous studies [1]. The literature reports that fat concentration in colostrum ranges from 5% to 8% between early and late colostrum, and that this may due to several factors, such as sampling time, type of diet and breed [1,29]. In our study the highest fat content was found at T24 and its overall evolution was consistent with previous reports [3].

The synthesis and secretion of lactose, a major osmotic component of sow’s milk, determines milk yield, which is consistent with the lower lactose concentrations observed in colostrum compared to mature milk by Craig et al. [29]. Our results showed an increasing trend in lactose levels from birth to 24 h postpartum, in agreement with Theil et al. [3], Klobasa [5] and Beyer et al. [25]. Total NFS in colostrum was affected by sampling time, reflecting the values obtained for the lactose and protein contents.

Mammary secretions in the first 24 h after the onset of parturition are rich in protein, especially in colostrum from the earliest phase. Colostrum proteins provide the neonate not only with a supply of amino acids but also a wide range of bioactive factors, such as hormones, cytokines, enzymes, growth factors, oligosaccharides and fatty acids [1,27,35]. Contrary to lactose and fat, the protein content decreased by about 50% between the first hours post farrowing, which is consistent with the findings of others [1,30] and followed the same pattern as immunoglobulin concentrations during the transition from colostrum to milk [17,29].

Unlike the effect of parity, the stage of lactation had a strong effect on colostrum composition, with large changes occurring in just a few hours.

Neonatal piglets are agammaglobulinemic at birth because the immunoglobulins circulating in sows cannot cross the placental barrier [17]; thus, the only way to acquire maternal antibodies is through the consumption of colostrum and the absorption of immune components through the gastrointestinal tract [36]. The concentration of IgG in colostrum is several-fold higher than in sow plasma [17]. In the present study, the highest immunoglobulin concentration in the total sera contents of the sows at the end of gestation was IgG (66%), followed by IgM (29%), both regardless of parity, and rather close to the values observed by Klobasa et al. [37], while the IgA concentration had the lowest values in the total sera contents of sows (5%) and was more likely to vary with parity. Sows with five or more parities had the highest concentration of IgA in their blood sera, although the only statistical difference was found between the fifth and fourth parities sows since the latter showed the lowest total concentration of immunoglobulins. By contrast, serum IgG was not affected by parity. This contrasts with the findings of Carney-Hinkle et al. [15] and Decaluwé et al. [32], who indicated that both serum IgG and serum IgA concentrations at the end of gestation were unaffected by sow parity. Although these authors did not study IgM levels, we found that parity had no effect on this immunoglobin. Corroborating these observations, Klobasa et al. [37] suggested an independent regulation of serum Ig levels, as they showed the independence among the three classes of Ig with regard to changes in their concentrations during the reproductive cycle of the sow. They suggested that the passage of serum Ig to the mammary gland could be different according to the isotype, although this hypothesis requires further study.

The immunoglobulin composition of sow colostrum contains approximately six times more IgG, IgM and IgA than sow milk [3]. IgG is the main immunoglobulin in colostrum, while IgA predominates in milk [5], as seen in this study, where IgG made up 86% of the Ig content of colostrum. Previously reported data for the effect of parity on the concentration of immunoglobulins in sow colostrum are inconsistent. Primiparous sows may have lower levels of immune factors (total Ig) than multiparous sows because the older sows have received more immune stimulation in the form of vaccinations or exposure to pathogens [38]. However, IgG concentration in the colostrum of sows has been reported to vary greatly [23,36,37] even among sows of the same unit [13].

Colostrum IgG concentration was not affected by parity, which is consistent with recent studies [21,29]. However, the lowest levels were observed in primiparous, second and fourth parity sows, although the differences were not statistically significant [21,30]. In addition, our results are partially consistent with those of the authors who observed that primiparous sows had similar IgG concentrations to those of second and fourth parity sows [8], although these authors observed large inter-farm differences. On the other hand, Quesnel [10] showed than the IgG concentrations were significantly influenced by parity at 24 h postpartum, but not at the onset of farrowing. In short, although some studies point to a parity effect on IgG concentration, there are many other and more powerful factors that affect their concentration.

In our study, colostrum IgA was not affected by sow parity. Although there has been very little research into the effect of parity on colostrum IgA, Carney-Hinkle [21] only observed a tendency for IgA concentrations to be greater in samples of colostrum and milk obtained from fourth parity sows compared with those obtained from primiparous animals. IgA is the major class of immunoglobulin found in sow’s milk and mucosal secretions and its importance lies in the control of pathogenic bacteria at the level of mucosal surfaces [1].

Our study revealed no effect of parity on IgM concentration. We are not aware of parity studies on this Ig, probably because IgM is a minor component of both colostrum (4%) and milk (18%) immunoglobulins [39], making it the least studied. However, IgM is important because it is the immunoglobulin that initially appears when an organism is exposed to an antigen for the first time [40]. Regardless of this, immunoglobulins in colostrum represent a history of the mother’s exposure to antigens and the response of her immune system. Indeed, it has been described that herd management (nutritional changes, genetic advances or timing of collection of samples) have a strong effect on the concentration of Ig in colostrum [8,29].

The highest concentrations of IgG, IgA and IgM in colostrum occurred immediately after farrowing, but decreased by approximately 45% within 24 h post-farrowing. After the onset of parturition, the IgG content in colostrum declined exponentially, while the IgA and IgM content only dropped slightly, which reflects its previously reported behavior [1,5,17]. The opposite relationship between lactose and immunoglobulins that was observed in this study has been described previously [30]. The strong differences observed in Ig concentration in just a few hours, following the onset of farrowing, emphasizes the importance of colostrum consumption by piglets as early as possible to acquire optimal colostral immunity.

A strong relationship was also found between serum IgG concentrations in the sow and colostral IgG and IgM content, but we found no significant correlation between IgG and IgA or between IgM and IgA, which concurs with an earlier conclusion reached by Markowska-Daniel [41]. Bourne and Curtis [9] observed that all calostral IgG, and a high proportion of IgM, appear to be derived from the maternal blood, unlike IgA, which is mainly synthesized in the mammary gland. Moreover, Quesnel et al. [29] concluded that IgG concentrations in maternal blood in late pregnancy explained the 36% variability observed in IgG concentrations in colostrum at the onset of farrowing. The sharply decreasing levels of IgG and lgM observed at the end of gestation would explain the transition of these immunoglobins from serum to mammary gland before farrowing [21,37]. By contrast, IgA levels sharply increased at the end of gestation, so that changes in the serum concentration of IgA during gestation display a very different pattern from IgG and IgM, as mentioned by other studies [21,37]. When milk secretion commences, the serum-derived IgG is supplanted by IgA synthesized in the mammary gland, which becomes the predominant Ig in milk [42]. The change in predominant immunoglobulin from IgG to IgA in a short period reflects the need of the piglet to acquire IgG for passive humoral immune protection in the first 24 h of life and then continuing protection from IgA in milk at mucosal surfaces [43]. These results provide evidence that IgG and IgM behave differently from IgA in sow colostrum, probably in relation to the origin of their synthesis. IgG comes almost exclusively from the sow’s serum, whereas IgA is mainly synthetized in the mammary gland.

## 5. Conclusions

This study shows that the reproductive parameters of sows were hardly affected by the number of parturitions, and only the birth weights of piglets born alive were lower in sows of first, third and fifth farrowing. Colostrum from primiparous sows contained higher concentrations of dry matter, fat, lactose and non-fat solids than those found in the colostrum from the rest of the sows, all of which produced colostrum that was nutritionally similar. This is important in order to carry out piglet adoptions in the early postpartum hours. By contrast, colostrum immunoglobulins were not affected by sow parity. We observed a strong effect of the lactation state on colostrum composition—the first colostrum collected being higher in protein and lower in lactose. Furthermore, the results provide evidence of the different behaviors of Ig in colostrum, especially those of IgG and IgM, with respect to IgA.

## Figures and Tables

**Table 1:** Influence of parity number on body condition and gestation length of sows and litter parameters at birth.

Item	Paritynumber					SEM	*p*-Value
	1	2	3	4	≥5		
Backfat thickness ^1^, mm	16.5	21.6	20.4	15.9	16.1	0.818	0.086
Loin depth ^1^, mm	46.9	42.7	44.5	41.9	40.1	1.118	0.321
Body condition score ^1^	2.8	2.7	3.1	2.8	2.6	0.086	0.588
NBA ^2^	13.0	11.0	12.5	13.3	12.7	0.451	0.541
NBD ^3^	0.7	2.2	2.0	0.9	1.8	0.237	0.157
LBW ^4^, kg	17.4	19.4	18.0	20.8	19.6	0.629	0.443
PBW ^5^, kg	1.3 ^b^	1.6 ^a^	1.4 ^ab^	1.5 ^a^	1.4 ^ab^	0.031	0.034
Gestation length, days	114.5	115.0	114.5	114.8	114.4	0.128	0.538

^1^ Values of back fat and loin depth and body condition for each sow at 110 day of gestation; ^2^ NBA, number of piglets born alive per litter; ^3^ NBD, number of piglets born dead per litter; ^4^ LBW, total litter birth weight; ^5^ PBW, birth weight of piglets born alive. ^a–b^ Means within the same row with different superscripts differ significantly at *p* < 0.05.

**Table 2:** Effect of parity number and sample collection time on the chemical compositions of colostrum from Large-White × Landrace sows.

Item	Parity Number					Time (h) ^1^			SEM	*p*-Value		
	1	2	3	4	≥5	0 ^1^	6	24		Parity	h	P × h
DM ^2^, %	28.1 ^a^	25.5 ^b^	23.9 ^b^	24.3 ^b^	25.2 ^b^	29.3 ^a^	24.0 ^b^	22.9 ^b^	0.351	0.001	<0.001	0.454
Protein, %	14.6	13.6	12.8	12.5	14.0	18.3 ^a^	13.5 ^b^	8.7 ^c^	0.293	0.150	<0.001	0.625
Fat, %	8.0 ^a^	7.0 ^ab^	6.1 ^b^	6.9 ^ab^	6.1 ^b^	6.1 ^b^	5.5 ^b^	8.8 ^a^	0.223	0.029	<0.001	0.505
Lactose, %	3.9 ^a^	3.3 ^b^	3.5 ^b^	3.6 ^ab^	3.5 ^b^	3.0 ^c^	3.5 ^b^	4.2 ^a^	0.065	0.024	<0.001	0.441
NFS ^3^, %	20.1 ^a^	18.5 ^b^	17.8 ^b^	17.4 ^b^	19.0 ^ab^	23.1 ^a^	18.5 ^b^	14.1 ^c^	0.268	0.015	<0.001	0.469
SCC ^4^, (×10^3^/mL)	5217	5718	3398	9397	2117	4229	5132	6139	1.069	0.323	0.742	0.447

^1^ Colostrum samples obtained at 0 h (immediately after the birth of the first piglet), 6 and 24 h. ^2^ DM, dry matter. ^3^ NFS, non-fat solids. ^4^ SCC, somatic cell count. ^a–c^ Means within the same row with different superscripts differ significantly at *p* < 0.05.

**Table 3:** Effect of parity number on IgG, IgM and IgA sera concentrations in Large-White × Landrace sows at 110 d of gestation.

Item	Parity Number					SEM	*p*-Value
	1	2	3	4	≥5		
IgG (mg/mL)	18.4	20.6	23.4	18.9	27.9	1.361	0.195
IgM (mg/mL)	10.4	9.6	10.4	8.6	9.6	0.416	0.610
IgA (mg/mL)	1.5 ^ab^	1.3 ^ab^	1.3 ^ab^	1.0 ^b^	2.3 ^a^	0.123	0.045

^a–b^ Means within the same row with different superscripts differ significantly at *p* < 0.05.

**Table 4:** Effect of parity number and time since farrowing on IgG, IgM and IgA concentrations in Large-White × Landrace sow colostrum.

Item	Paritynumber					Time (h) ^1^			SEM	*p*-Value		
	1	2	3	4	≥5	0	6	24		P	H	P × h
IgG (mg/mL)	63.3	62.3	76.4	63.1	71.8	94.3 ^a^	70.6 ^b^	37.3 ^c^	1.891	0.349	<0.001	0.398
IgM (mg/mL)	4.6	4.4	3.7	4.9	4.9	5.0 ^a^	4.7 ^a^	3.7 ^b^	0.159	0.201	0.005	0.947
IgA (mg/mL)	8.1	9.1	7.4	7.4	6.5	9.9 ^a^	8.2 ^b^	5.1 ^c^	0.233	0.167	<0.001	0.978

^1^ Colostrum samples obtained at 0 h (immediately after the birth of the first piglet), 6 and 24 h. ^a–c^ Means within the same row with different superscripts differ significantly at *p* < 0.05.

**Table 5:** Correlation coefficients between serum and colostrum immunoglobulin (IgA, IgG and IgM) content of sows ^1^.

	Serum IgA	Serum IgM	Colostrum IgG	Colostrum IgA	Colostrum IgM
Serum IgG	0.006	0.082	0.571 ***	−0.063	0.438 **
Serum IgA		0.072	0.087	0.159	0.098
Serum IgM			0.007	−0.215	0.013
Colostrum IgG				0.115	0.995 ***
Colostrum IgA					0.184

^1^ Colostrum Ig concentration averages at 0, 6 and 24 h; ** *p* < 0.01; *** *p* < 0.001.
